# Tackling the Challenging Determination of Trace Elements in Ultrapure Silicon Carbide by LA-ICP-MS

**DOI:** 10.3390/molecules28062845

**Published:** 2023-03-21

**Authors:** Davide Spanu, Alessandro Palestra, Veronica Prina, Damiano Monticelli, Simone Bonanomi, Sandro Usseglio Nanot, Gilberto Binda, Laura Rampazzi, Gianluca Sessa, David Callejo Munoz, Sandro Recchia

**Affiliations:** 1 Department of Science and High Technology, University of Insubria, Via Valleggio 11, 22100 Como, Italy; 2 SiCreate GmbH, Via Piave 6, 23871 Lomagna, Italy; 3 Norwegian Institute for Water Research (NIVA), Økernveien 94, 0579 Oslo, Norway; 4 Department of Human Sciences and Innovation for the Territory, University of Insubria, Via Sant’Abbondio 12, 22100 Como, Italy; 5 Dipartimento di Scienze della Terra, Università degli Studi di Milano, Via Luigi Mangiagalli 34, 20133 Milan, Italy

**Keywords:** silicon carbide, laser ablation, ICP-MS, microwave-assisted acid digestion, trace elements, LA-ICP-MS, direct analysis

## Abstract

The goal of accurately quantifying trace elements in ultrapure silicon carbide (SiC) with a purity target of 5N (99.999% purity) was addressed. The unsuitability of microwave-assisted acid digestion followed by Inductively Coupled Plasma Mass Spectrometry (ICP-MS) analysis was proved to depend mainly on the contamination induced by memory effects of PTFE microwave vessels and by the purity levels of acids, even if highly pure ones were used in a clean environment. A new analytical protocol for the direct analysis of the solid material by laser ablation coupled with ICP-MS (LA-ICP-MS) was then exploited. Different samples were studied; the best results were obtained by embedding SiC (powders or grains) in epoxy resin. This technique has the great advantage of avoiding any source of external contamination, as grinding, pressing and sintering pretreatments are totally unnecessary. Two different laser wavelengths (266 and 193 nm) were tested, and best results were obtained with the 266 nm laser. The optimized protocol allows the determination of elements down to the sub-mg/kg level with a good accuracy level.

## 1. Introduction

Silicon carbide (SiC) is a deeply studied semiconductor and it is increasingly used to produce advanced ceramics thanks to its exceptional physicochemical properties, such as being lightweight, chemically inert, having a wide bandgap and outstanding magnetic, thermal, mechanical, optical and electronic properties [1,2]. Given these features, SiC finds application as a major component of materials in a variety of industrial sectors, such as semiconductors, cutting materials, grinding materials, high-temperature energy conversion systems, nuclear reactors, and fusion reactors [3,4,5]. However, the purity of SiC is becoming a critical factor for the development of new and highly technological applications (e.g., photovoltaics, electronics). As a matter of fact, ultrapure SiC (99.999% pure, 5N) is desirable in these fields as its properties are significantly influenced by defects and impurities, even when present at the ultratrace level [6,7,8]: increased thermal conductivity, decreased electrical resistivity and enhanced resistance to chemical erosion are achieved only when using highly pure SiC materials. Trace contaminants may adversely affect the efficiency of SiC-based devices; this is why their accurate determination in SiC is key for the design of new and innovative technologies (e.g., electric mobility).

In this context, several analytical techniques have been applied [9,10,11,12,13,14,15]. The most common approach involves the dissolution of the sample followed by inductively coupled plasma mass spectrometry (ICP-MS) or ICP-optical emission spectrometry (ICP-OES) analysis [6,16,17,18,19,20]. The need of samples in solution form is, however, a major limitation when treating SiC ceramics: SiC is extremely inert and difficult to dissolve even under extreme conditions (e.g., high temperatures, presence of concentrated acids and bases). This process is, in fact, typically carried out under highly harsh conditions by microwave acid digestion (e.g., H_2_SO_4_ + HNO_3_ + HF at 230 °C for 20 h [16] or even at higher temperatures up to 250 °C [20]) or alkaline melting (e.g., Na_2_CO_3_ + Na_2_B_4_O_7_ or Li_2_B_4_O_7_ in platinum crucibles at 1000 °C for 30 min [17]). Nonetheless, it should be pointed out that (i) dedicated equipment is required to carry out these extreme dissolution protocols; (ii) the use of alkaline fusion is very limited for the determination of ultra-trace impurities because of the high background signal generated by the significant saline content in solution; (iii) microwave acid digestion may suffer from high blank signals due to the limited purity of the acid mixture (especially that of sulfuric acid, which is essential because of the high temperatures that need to be reached) making the determination of trace elements such as Cu, Fe and Al difficult [16]. Owing to these drawbacks and the dilution performed when using these strategies, the detection limits that are generally reported for these elements are higher than mg/kg levels [21,22,23], questioning the adoption of wet digestion methods for the determination of ultra-trace impurities.

Parallel to these methods, direct analytical techniques are studied as valid alternatives to overcome the above-mentioned limitations; these include instrumental techniques that work directly on the solid sample, which have the advantage of not requiring sample pretreatment, thus limiting potential sources of contamination and making the analytical protocol simpler and “greener”. In this framework, glow discharge mass spectrometry (GD-MS) is generally preferred over other techniques for the direct determination of trace elements in semiconducting materials because of its high sensitivity, limited matrix effect and reduced risk of contamination [24,25,26]. In fact, detection limits in the order of tens or hundreds of µg/kg are commonly reported for GD-MS measurements with a dynamic linear range extended over more than 10 orders of magnitude [27,28,29]. Even though GD-MS represents a valuable solid-state method, its very high cost (e.g., the need of high mass resolution to overcome spectral interferences) may inhibit its adoption for the high-throughput analysis of numerous samples (e.g., routine quality control analysis on SiC production batches). Attempting at filling this gap, the laser ablation technique coupled with ICP-MS (LA-ICP-MS) has been recently proposed as a simpler and cheaper alternative direct analytical method for the determination of trace elements in SiC materials; as a matter of fact, the instrumental cost of LA-ICP-MS is at least four times lower than that of double-focusing GD-MS. LA-ICP-MS provides reliable quantitative information down to mg/kg and sub-mg/kg levels over a dynamic linear range of 4–5 orders of magnitude [30]. However, only a very poor number of works have reported this technique [16,30,31,32] and no clear current trends can be defined. Notwithstanding the promising analytical performances, many critical issues should be tackled to make LA-ICP-MS a reliable method suitable for the determination of trace elements in SiC; as an example, universal sample preparation (i.e., physical immobilization/stabilization of powdered samples) and calibration strategies (necessarily matrix-matched) are not yet conceived. The first point may be particularly challenging: as of today, the only methods that are proposed in the literature include laborious and multi-step sample pretreatments involving, for instance, the thermal sintering of SiC powders at 1000 °C [16]. Such an approach may lead to significant sample contamination or alteration owing to sample manipulation. As is clearly shown in this work, it is absolutely not trivial to pass from the analysis of 99% pure SiC to the analysis of 99.999% pure SiC. In this respect, none of the methods proposed in the literature (other than GD-MS) could be used for ultrapure SiC and this gap should be filled to provide a new reliable analytical tool for the development of future high-quality SiC-based technologies.

Given the current analytical problems, we addressed the goal of quantifying trace elements in ultrapure SiC (99.999% purity, i.e., the sum of all impurities cannot exceed the concentration of 10 mg/kg) by first investigating the potentialities and demonstrating limits of microwave acid digestion followed by ICP-MS analysis and then by developing a new analytical protocol for the direct analysis of the solid material with LA-ICP-MS; two different sample preparation strategies were critically evaluated.

## 2. Results and Discussion

As stated in the introduction, we preliminarily exploited wet digestion methods followed by ICP-MS analysis. We found that this strategy was not applicable for the detection of impurities in ultrapure SiC due to the insufficient purity level of highly pure acids (especially H_2_SO_4_) and the contamination of samples caused by the harsh mineralization conditions. For the interested readers, this is extensively detailed in the Supporting Information (Figures S1 and S2 and Table S1).

### 2.1. LA-ICP-MS Analysis

Laser ablation represents a good alternative to GD-MS because very low background signals are normally recorded (when the sample is placed under He flow without performing its ablation) and because the solid sample is analyzed as it is, i.e., without any dilution. Nevertheless, one of the fundamental requirements for a good quantitative analysis with laser ablation is that the sample must be as compact and planar as possible so that the laser beam hits perpendicularly and favors a better and more reproducible ablation of the material. While it is easy to understand that the laser ablation efficiency varies if the beam does not hit the sample perpendicularly, the need of working with compact samples requires an in-depth discussion. As we show later, the energy of laser shots could be either used (wasted) to lift particles from the ablated surface or (better) to sublimate the sample. In this respect, powdered samples are not suitable as they are because the lifting of particles is dominant. This effect causes a strong instability in the analytical signal together with a lower sublimation efficiency. Therefore, such powdered samples should be pretreated to have a somewhat enhanced degree of compactness. To face this problem, we decided to use two different strategies: the fabrication of binder-free SiC tablets (as proposed by Zhou et al. [16]) and the embedding of SiC powders in epoxy resin (newly developed strategy).

#### 2.1.1. Fabrication of Binder-Free SiC Tablets and LA-ICP-MS Analysis

The first attempts to produce binder-free SiC tablets were carried out by pressing grinded SiC powder (finely grinded in an agate mortar) for two hours using a pressure equal to 9 × 10^3^ kg/cm^2^. However, we were not able to obtain a sufficient degree of compactness, the resulting tablet being extremely brittle and characterized by the presence of non-aggregated particles. This is one of the main criticisms of using an agate mortar: although it is widely used in the literature to pulverize SiC, ceramics and refractory materials [33,34,35], it does not possess a sufficient hardness to efficiently generate small SiC particles (Mohs scale values 6.5–7 and 9 for agate and SiC, respectively [36,37]). As an additional drawback, sample contamination may occur during the grinding process. With the aim of evaluating the suitability of using a conventional agate mortar, we decided to attempt to increase the compactness of SiC tablets to make them applicable for LA-ICP-MS. More specifically, we followed a procedure reported in the literature involving a thermal treatment at 1000 °C for 2 h of the pressed SiC grinded powder to induce the partial sintering of the material [16] (Figure S3 shows that no changes occurred in the crystalline features of SiC sample after the thermal treatment). With such a treatment, the tablet was sufficiently compact to be analyzed with LA-ICP-MS.

The main ablation parameters, i.e., the spot diameter and the beam power, were then optimized to avoid the excessive lifting of dusty material and to maximize the sublimation efficiency. It emerged that high fluences and large spots led to micro-explosions (detachment of dust grains from the surface near the ablated area) and consequently to the potential entrainment of excessively large material in the ICP torch.

Using a line ablation pattern (scan speed = 40 μm/s, ablation frequency = 10 Hz), a sufficient compromise was found with a 40 μm spot size and a fluence equal to 2.44 J/cm^2^. Any increase of fluence or spot size dramatically increases the powder lifting, which is already quite effective even in optimized conditions, as can be clearly seen in Figure S4.

**Figure 1:**
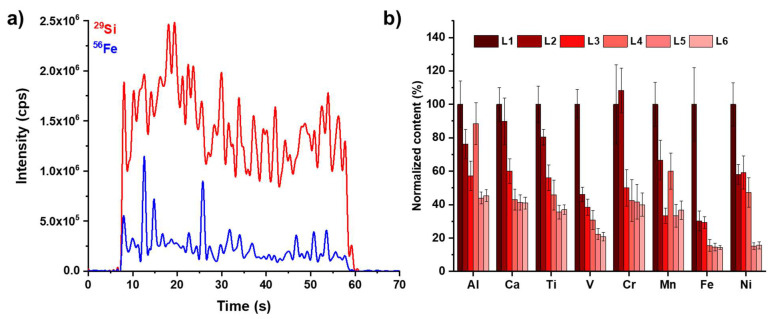
(**a**) Examples of ablation signals over a 2 mm long line and (**b**) depth profile analysis of the selected elements. Both measurements were carried on the standard reference material BAM S003a: the relative amounts of trace elements reported in (**b**) are determined using ^29^Si as the internal standard.

An example of the obtained signals using the above-reported conditions is depicted in Figure 1a.

LA-ICP-MS quantitative analysis was carried out by ablating and averaging three different lines, each one ablated five times (the “Ln” labels refer to the nth passage of the laser beam on the same coordinates of the sample): the first four lines (L1, L2, L3 and L4) were not considered for quantitative purposes because the material ablated from these zones may be affected by external contamination because of all sample manipulations (e.g., exposure to the atmosphere, handling, pressing into tablets). This cleaning strategy exploits one of the numerous advantages of LA-ICP-MS, that is, the possibility to eliminate the surface contamination. It should be noticed that analogous benefits are also achieved with GD-MS. We decided to use a five-line ablation pattern for all analyzed samples, obviously considering only the fifth line for quantitative purposes since for all the eight selected elements (Al, Ca, Ti, V, Cr, Mn, Fe and Ni) analyzed, the fifth and the sixth lines obtained over the standard reference material yielded stable results (see Figure 1b).

Once the laser parameters and the sampling pattern were defined, we performed a quantitative determination of trace elements in two SiC samples (named SiC-1 and SiC-2) using the standard reference material BAM S003a as the calibrating material. The results were compared with those obtained by GD-MS (see Table 1); it is worth recalling that GD-MS was considered as a reference technique in this work. To be confident with this assumption, we decided to perform a blind analysis of the BAM S003 reference material by external GD-MS; the obtained results clearly indicated (see Table S2) that GD-MS provided accurate and precise data.

As a first observation, most trace elements were largely overestimated. This was especially true for those elements affected by contamination during pretreatments (e.g., Fe, Ni and Ca, potentially deriving from the systems used for grinding and pressing SiC into tablets). Additionally, signals were affected by a very low and insufficient precision (relative standard deviation also up to 50% for elements with concentrations higher than 5 mg/kg). Any attempts to improve the quality of these data by using the ^29^Si signal as the internal standard were unsuccessful.

The occurrence of contamination, which is beyond doubt, prompted us to radically change the sample pretreatment, rather than to further optimize the ablation conditions; please note that the contamination mainly derives from the grinding of SiC in the agate mortar, which induces a bulk contamination that cannot be lowered by preventive ablation cleanings.

#### 2.1.2. Embedding of SiC Powders in Epoxy Resin and Preliminary Observations

Owing to the above-mentioned (and verified) drawbacks connected to already-reported strategies [16], we decided to develop a new approach based on embedding the SiC samples in epoxy resin without performing any mechanical or thermal pretreatments. In principle, embedding should ensure better mechanical stability and enable overcoming both the problems related to the contamination and fragility of the sample, with the latter one being the main reason for the observed excessive grain lifting.

Both grains and fine powder SiC samples were embedded in epoxy resin using the procedure reported in the experimental section. SiC surfaces were exposed thanks to proper cutting and polishing operations; after such procedure, the samples were externally cleaned by pickling with HCl 0.1 M. As the result, the embedded materials were highly compact, regardless of their morphological nature (see Figure S5) and could be handled without special precautions, as is necessary for binder-free SiC tablets.

During the optimization of the ablation parameters, we immediately observed the total absence of grains or powder liftings, even when working with the largest available spot size (120 µm) and increasing the fluence from 2.44 to 3.8 J/cm^2^. This is a symptom of a significantly increased mechanical stability. As a preliminary observation, the total absence of the unwanted powder lifting (possibly transported to the ICP-MS) ensured much more stable and less noisy signals. Moreover, the larger spot size, together with the higher fluence, allowed an improvement of more than an order of magnitude in terms of instrument sensitivity: this is mainly due to the nine-fold increase of the ablated surface obtained when passing from the 40 µm to the 120 µm spot.

**Figure 2:**
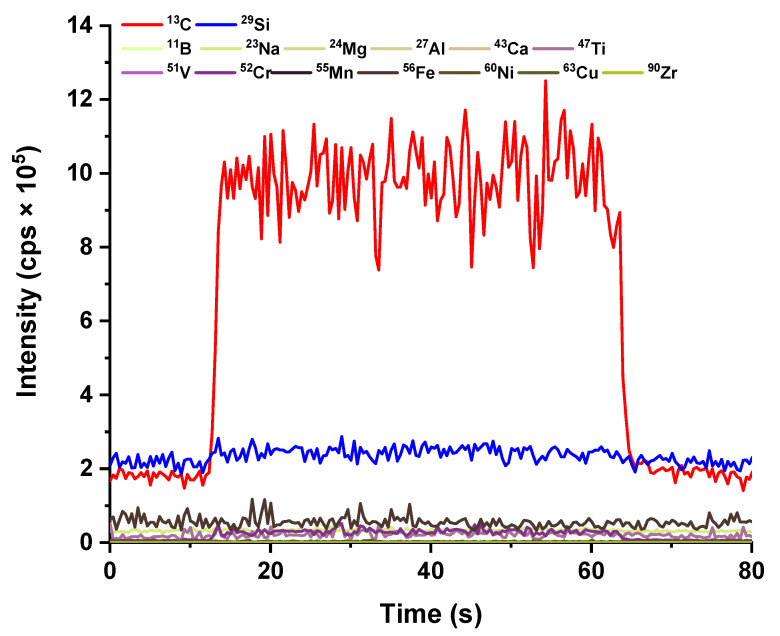
Ablation signals recorded by analyzing the epoxy resin used in this study. Spot size = 120 µm, length = 1 mm, scanning speed = 20 µm/s, frequency = 10 Hz and fluence = 3.8 J/cm^2^.

The evaluation of the trace element content in the employed epoxy resin was necessary to assess eventual contaminations induced by the resin itself. This step was mandatory before we could start developing the overall analytical protocol. The signals recorded during the ablation of a resin-only surface are depicted in Figure 2 and demonstrate that the concentration of practically all investigated elements was below the limit of detection of the technique (the ablation signal was not statistically different from the background signal for most of the elements). The only isotopes appreciably detectable in the resin matrix were ^13^C and ^29^Si; the first one is not an element of interest for the analysis, whereas ^29^Si produced a signal barely distinguishable from the background (in any case greatly lower than the ^29^Si signal obtained by the ablation of SiC materials). In conclusion, this embedding approach can be safely used, as no sample contamination is induced by the utilization of the epoxy resin.

**Figure 3:**
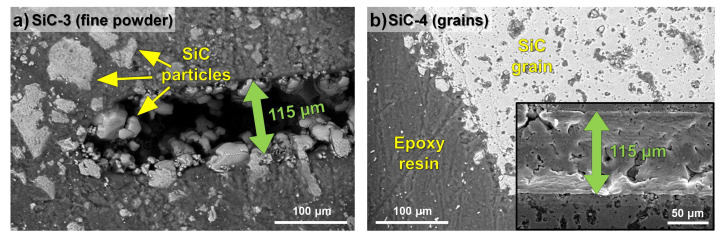
SEM images of craters generated by laser ablation lines on samples (**a**) SiC-3 and (**b**) SiC-4.

The effect of the granulometry of SiC samples on the morphological features of the craters being generated by laser ablation lines is shown in Figure 3.

As a first observation, the fine-powdered SiC (Figure 3a) was not evenly distributed in the epoxy resin: most of the surface seemed to be covered with the resin, containing micrometric-sized SiC grains. In contrast, granular materials (Figure 3b) were intrinsically compact and more uniform. The main consequence was that the ablation efficiencies found for powder and granular SiC samples seemed largely different. Apart from the width of the ablation (around 115 µm for both the materials), significant differences were found concerning the depth and the aspect of the dig. In more detail, a much deeper ablation was obtained for the fine powder (SiC-3, 150–250 µm) compared with the granular material (SiC-4, 35 µm). The latter also appeared to be the subject of a surface melting process due to the very high temperature locally reached under the laser beam.

**Figure 4:**
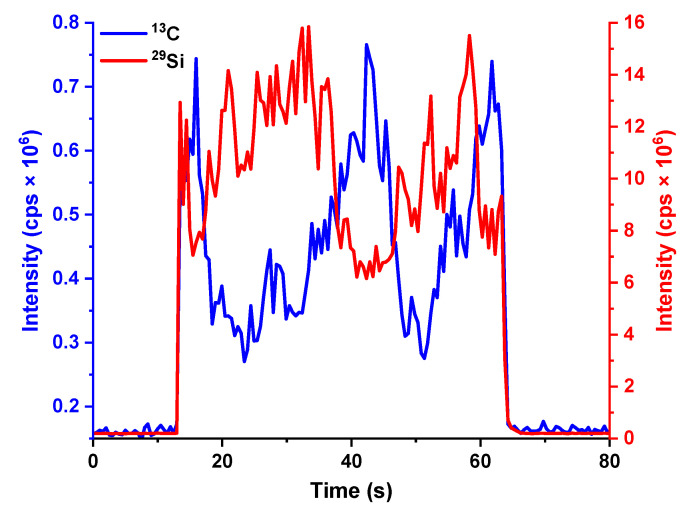
^13^C and ^29^Si ablation signals recorded by analyzing SiC-3. Spot size = 120 µm, length = 1 mm, scanning speed = 20 µm/s, frequency = 10 Hz and fluence = 3.8 J/cm^2^.

For the fine powder sample SiC-3, these morphological observations were well in line with the inhomogeneous signals recorded by LA-ICP-MS analysis (Figure 4): significant signal fluctuations were observed for Si, which apparently has a complementary profile with respect to the ^13^C signal. This evidence suggests that SiC tiny grains and the resin (mostly made of carbon) are alternatively ablated along the 1 mm line owing to the inhomogeneous distribution of SiC particles in the resin. The same behavior was not observed for granular samples.

To solve this issue, two main improvements were applied. Firstly, since line analysis works perfectly when granular samples are studied but may lead to data misinterpretation when fine powders are analyzed, line analysis was replaced by spot analysis. Secondly, before the resin embedding, SiC powders were compacted as much as possible to strongly increase the SiC/resin surface ratio.

**Figure 5:**
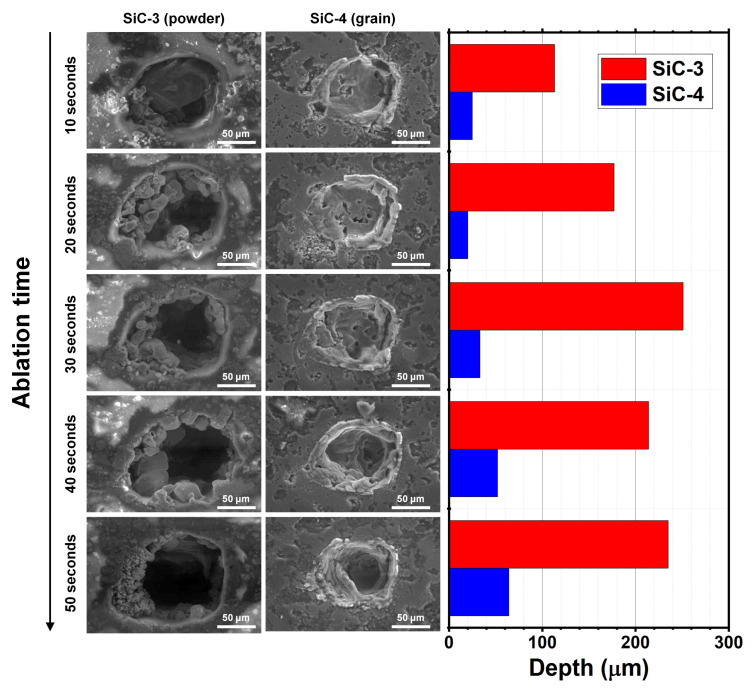
SEM images of craters generated by laser ablation spots on samples SiC-3 and SiC-4 using different ablation times. The bar plot shows the depth of the craters for both samples as measured by SEM.

On these bases, we investigated the morphology of craters generated by spot ablation on SiC-3 and SiC-4. As shown in Figure 5, the holes produced by consecutive ablation on the same spot (spot size 120 µm, five consecutive ablations lasting for 10 s with a frequency of 10 Hz) on fine powder and granular samples were still significantly different. Analogously to the 1 mm long line ablation, a much deeper crater was obtained for the fine powder (up to 250 µm) compared with the granular material (up to 60 µm). The maximum depth was apparently reached already after 30 s (three consecutive ablations lasting for 10 s) for the powdered sample SiC-3, whereas the depth of the crater for the granular material (SiC-4) also slightly increased after three consecutive ablations. Moreover, no significant statistical differences were found when comparing LA-ICP-MS signals obtained from the third and fifth ablation series (both for fine powder and granular samples), meaning that two consecutive ablations are sufficient to “clean” the sample from surface contamination and achieve reliable results. This information was used to define the final version of the analytical protocol as discussed in the following paragraph.

#### 2.1.3. Analytical Performance of the Developed LA-ICP-MS Analytical Protocol

According to the evidence reported in the previous paragraphs, we defined the final version of the analytical protocol as follows.

**Figure 6:**
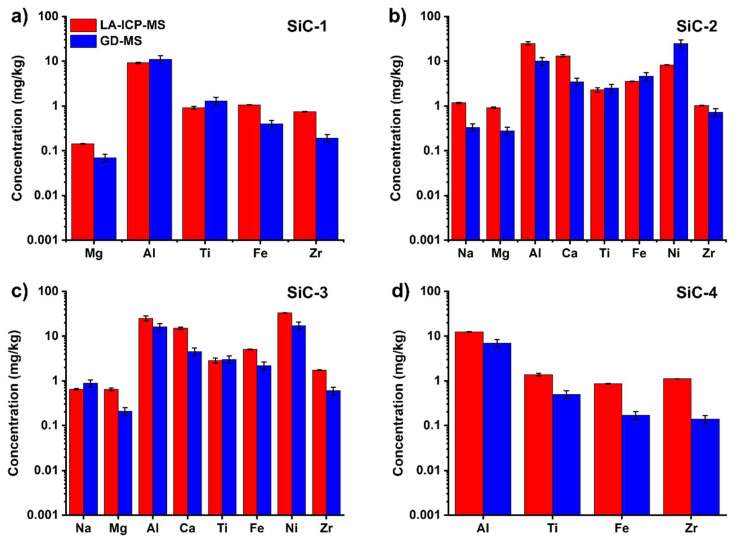
Concentration of selected trace elements in SiC samples as determined by LA-ICP-MS (red bars) and GD-MS (blue bars): (**a**) SiC-1, (**b**) SiC-2, (**c**) SiC-3 and (**d**) SiC-4. Elements not included in this Figure for specific samples are below the detection limits of LA-ICP-MS (see details in Table S4).

SiC samples embedded in epoxy resin were treated with three consecutive spot ablations (120 μm, 3.8 J/cm^2^, 10 Hz, 10 s) in five different zones of the sample surface: the first two ablations were used to “clean” the sample surface from external contamination, while the third one was analyzed for quantitative purposes. Such approach led to a significant reduction in analysis time: the analysis of each spot required less than one minute considering the cleaning of the surface, laser warm-up and sample holder movement steps. Four different SiC samples with different impurity types and levels were analyzed using the standard BAM S003a as the calibrant and using the signal of ^29^Si as the internal standard. Of course, only elements resulting as higher than the limit of detection of GD-MS and certified in BAM S003a were considered. The results are depicted in Figure 6.

We preliminarily selected a limited number of elements (those sufficiently abundant in the samples to be detected by GD-MS, see Table S3, and certified for BAM S003a). This choice was also made in consideration of the limited duration of the effective analysis (10 s for each spot). As can be observed in Figure 6 and Table S4, the concentrations determined by LA-ICP-MS were highly precise (relative standard deviation higher than 5% only for Ti and Al, see Table S4) and, more importantly, close to those determined by GD-MS, including for sub-ppm concentrations. Indeed, statistically significant differences were found for some elements but such errors could be reasonably ascribed to the facts that (i) concentrations close to sub-ppm levels represent the limit of detection of LA-ICP-MS [38]; (ii) the different granulometry and crystallographic features of the calibrant and the samples may account for a different ablation efficiency; (iii) the elemental concentrations in the calibrant BAM S003a were much higher than those in the samples (see Tables S1 and S2), leading to less accurate quantification of low levels and (iv) GD-MS quantification may itself be affected by errors. Concerning this final point, it is important to underline that external GD-MS clearly states that biases up to 20% should be considered acceptable if the sample matrix does not perfectly match the one of the standards. Concerning the granulometry, better results may be achieved by grinding and homogenizing the SiC sample prior to the embedding in the epoxy resin by using a mortar made of extremely hard materials (e.g., boron carbide), which should allow reducing the particle size without causing significant contamination (as instead occurs using an agate mortar). As a final consideration, bad agreement between GD-MS and LA-ICP-MS normally appears at low concentrations and for elements with quite low sensibility (as an example, the sensibility of Zr is only 1/10 that of Ti). With such extremely low signals, the mismatch between GD-MS and LA-ICP-MS is fully understandable. Nonetheless, it is beyond doubt that the information gained by the simple and fast LA-ICP-MS is reliable and reflects the results obtained by GD-MS analysis, providing a robust and important screening indicator of the purity of SiC materials (e.g., defining a 5N purity) and thus it can represent a much cheaper, simpler and faster alternative to GD-MS for routine and high-throughput applications; the laser ablation analysis of one sample requires around 5 min, including the analysis of the calibrant.

Proving the effectiveness of the method, the limit of detection (LOD) was calculated, according to previous literature [38], as three times the standard deviation of the gas blank divided by the calibration sensitivity. It should be noticed that the wide variability of the LODs reported in Table 2 is mainly ascribed to the extremely different background signal levels recorded on different m/z channels. In any case, the results summarized in Table 2 clearly show that sub-ppm levels could be reliably detected for practically all the elements by using the proposed LA-ICP-MS protocol. The reproducibility was assessed over three replicated sample analyses; a mean relative standard deviation (RSD%) of 4.0% was found considering all 13 selected elements with some critical elements such as Ti (9.7%) and Al (6.8%).

As a final optimization, we attempted to increase the ablation efficiency in two ways: (i) by increasing the laser fluence up to 6.0 J/cm^2^ and (ii) by changing the wavelength of the laser source. Concerning the first point, no significant effects on the sensitivity were found and, additionally, the signal appeared much noisier, making the detection of ultratrace elements harder; such behavior is reasonably ascribed to the excessively energetic radiation that favors the partial micro-explosions rather than the vaporization of the material.

**Figure 7:**
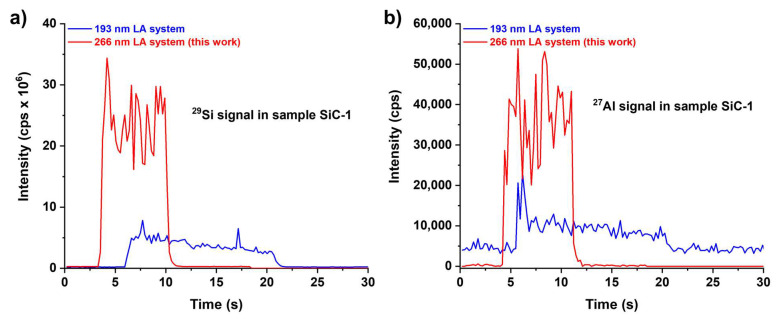
Signal profiles of (**a**) ^29^Si and (**b**) ^27^Al acquired by using an excimer-based 193 nm (blue lines) and a Nd:YAG-based 266 nm (red lines) LA-ICP-MS instrumentation.

Concerning the second point, the same SiC samples were analyzed under the same experimental conditions (spot size = 120 µm, frequency = 10 Hz, ablation time = 10 s and fluence = 3.8 J/cm^2^ and the same ICP-MS instrumentation) but using an ArF excimer-based 193 nm laser ablation system (see details in the Experimental section); it is in fact well known that increased ablation efficiencies are normally achieved with this laser type. However, as clearly visible in Figure 7, the signals acquired with the 193 nm laser ablation system appeared much broader, non-constant and markedly lower in terms of sensitivity, thus ruling out any significant beneficial effect induced by the utilization of the 193 nm laser rather than the 266 nm one (used in this work).

These apparently odd observations can be easily explained considering that the increase of sensibility expected for the 193 nm laser actually existed but was so small that it was hidden by the better geometry of the 266 nm laser ablation cell. In fact, although both systems were equipped with two-volume cells, the one mounted on the 266 nm laser was based on the viscous film chamber geometry, which allowed achieving much lower wash-out times than those obtained by commercial ablation chambers (e.g., the one equipped with the 193 nm laser ablation instrumentation), thus providing a strongly enhanced sensitivity [39].

## 3. Materials and Methods

### 3.1. Silicon Carbide Samples

Four silicon carbide samples (SiC-1, SiC-2, SiC-3 and SiC-4) and a reference standard material (BAM S003a, Bundesanstalt für Materialforschung und -prüfung, Berlin, Germany) were used in this work. Concerning the preparation of SiC samples, they were obtained by mechanical mixing of quartz powder with commercial graphite powders with different purities. The mixture was placed in a graphite crucible and heated by means of an induction furnace up to 1700 °C under a controlled atmosphere (Ar) and kept at the reaction temperature for about 30 min before subsequent cooling. The resulting yellow/green SiC powder was then heated in oxygen at 600 °C to remove unreacted graphite.

Reference trace element concentrations reported in Table S3 were determined by high-resolution GD-MS (Astrum HR-GD-MS from Nu Instruments, Clywedog Rd S, Wrexham LL13 9XS, Wrexham, UK). The GD-MS analyses were purchased from an external laboratory (Eurofins EAG Laboratories, Toulouse, France). Briefly, the procedure consists of a mechanical preparation (SiC samples broken into small pieces) with a resulting piece placed and pressed onto a pre-etched high-purity (7N+) Indium pin. The plasma conditions were applied for 10 min prior to taking any reading to perform the analysis in the bulk of the material. The analysis was conducted by cooling the glow discharge ion source to a temperature near that of liquid nitrogen. Each element was measured at least three times. For the sake of calibration, the RSF (Relative Sensitivity Factor), a factor applied on the ion beam ratios to calculate the concentration ratios, was estimated by analyzing a large quantity of certified and matrix-matched reference materials. The samples were analyzed, and each element was measured between 3 and 5 times. The average of these measurements was reported.

All the samples were also characterized in view of their crystallographic features by XRD analysis (Siemens D5000 XRD that uses Cu-Kα radiations, 40 mA, 40 kV with steps of 0.02° (2θ), scanning speed of 3 s/step, 1 mm slit and 25–65°, see Figure S6). Peak attribution was performed according to references in the literature [40].

### 3.2. Microwave-Assisted Acid Digestion

Microwave acid digestion was carried out with an ETHOS One instrument (Milestone MLS) equipped with a carousel of 10 vessels in polytetrafluoroethylene (PTFE, internal volume ~100 mL). For each digestion batch, 3 vessels were used for SiC samples and 2 for reference blanks. The adopted strategy initially involved the exploitation of the “Vessel-Inside-Vessel” technique [41,42] to avoid memory effects [43]; digestion took place in small capped containers of perfluoroalkoxy (PFA, internal volume = 5 mL) placed inside commercial PTFE vessels. The sample (mass approximately equal to 50 mg) was introduced into the PFA vessel together with the mixture of acids, while 5 mL of ultrapure H_2_O was placed outside of it (i.e., inside the PTFE vessel). A sample was always inserted into the vessel with the temperature probe to monitor and control the digestion process. The digestion was carried out in a mixture composed of 1.5 mL of H_2_SO_4_ (Analytika, ≥95%, Analpure™ for trace analysis), 1.5 mL of HF (Carlo Erba, Cornaredo, Italy, 47–51%, Ultrapure-for trace analysis) and 1.5 mL of HNO_3_ (obtained by distillation from commercial HNO_3_, Carlo Erba 65% [44]) at 230 °C for a maximum duration of 48 h. The digestion process follows the temperature program reported in Figure S7. The microwave power was automatically regulated to reach the set temperature and it was limited to 1000 W. The same conditions were also applied using the conventional microwave oven configuration (solid sample placed in the 100 mL PTFE vessel).

Once the digestion was completed, the samples and blanks were transferred into low-density polyethylene (LDPE) bottles and diluted to 30 g with ultrapure H_2_O (produced from a Sartorius Arium mini UV Lab Water system). They were then again diluted 1:10 (Rh was added as internal standard) and analyzed with a Thermo Scientific ICAP Q ICP-MS equipped with a He collision cell and kinetic energy discriminator (KED). Given the use of HF as an etching acid, it was necessary to equip the ICP-MS with a platinum injector and PFA spray chamber. The following elements were analyzed in KED mode: B, Na, Mg, Al, Ca, Ti, V, Cr, Mn, Fe, Co, Ni, Cu, Zn, Sr, Zr, Mo, Ag, Cd, Ba, Sn and Pb. ICP-MS working parameters are reported in Table 3.

Before their use, the LDPE bottles were decontaminated with a three-step process [42]: (i) washing with ultrapure H_2_O and immersion in a 0.4% *v*/*v* Nalgene L900 detergent solution for one week; (ii) rinsing with ultrapure H_2_O and placement in a 2% wt. HNO_3_ solution for one week and (iii) rinsing with ultrapure H_2_O and placement, for a week, in a second solution of 2% wt. HNO_3_. Finally, they were rinsed with ultrapure H_2_O before use.

The evolution of the morphology of any residual particles after acid digestion (i.e., undissolved particles) was verified by observations with the scanning electron microscope (ESEM FEG XL30, Philips, Amsterdam, The Netherlands).

### 3.3. Sample Pretreatment for LA-ICP-MS Analysis

For the direct analysis of the solid sample by LA-ICP-MS, it was necessary to make the sample compact and planar. To do this, two main protocols were applied:

1. Production of a SiC tablet: the sample was finely ground in an agate mortar and the obtained powder was pressed with a tablet press (Specac) to produce a compact SiC tablet (pressure = 9 × 10^3^ kg/cm^2^, 30 min). With the aim of improving its mechanical stability, the tablet was sintered at 1000 °C for 2 h (ramp rate 5 °C/min) [16]. After this operation, the tablet was analyzed by LA-ICP-MS;
2. Embedding in epoxy resin: the SiC sample without any mechanical pretreatment (i.e., in fine powder or grain form depending on the nature of the starting material) was embedded in an epoxy resin (Epofix Kit, Struers, Copenhagen, Denmark) as follows. The resin and the hardener were gently mixed with a volumetric ratio of 2:15 for 2 min, attempting to avoid the formation of air bubbles. Then, after 2 min of rest, the resin was poured on a small amount of sample previously placed (and eventually gently pressed) in the center of a cylindric mold until a cylinder of 3 or 4 cm was obtained. Immediately after resin pouring, the mold containing the sample and the fresh liquid resin was placed in a vacuum chamber to remove air bubbles and favor the penetration of the resin in SiC pores (pressure around 150 mbar for 15 min). After 24 h of hardening, the resin was cut into a 2.5 mm thin disc. If the sample was granular, a cut of about 750 µm was performed on the sample side to obtain a cross-section of exposed SiC grains. Then, the sample was polished with a 500-grit sandpaper and a pickling operation was applied in 0.1 M HCl for 45 min in a mechanical shaker at 130 rpm in order to remove any surface contamination due to sample handling.

### 3.4. LA-ICP-MS Analysis

The analysis of the samples was carried out using a laser ablation cell previously developed by our research group [39]. The used system combines a separate Laser Ablation System (UP 266, New Wave Research, Fremont, CA, USA) with a Thermo Fisher IcapQ ICP-MS (source Nd: YAG, 266 nm pulse 3–4 ns). The transport of the ablated material is guaranteed by a He flow of 1 L/min. The analyses were conducted by placing more than one SiC sample inside the cell: a certified standard material (BAM-S003a) was used as a single matrix-matched calibration standard.

The signals on the following m/z channels were collected: ^7^Li, ^11^B, ^13^C, ^23^Na, ^24^Mg, ^27^Al, ^29^Si, ^39^K, ^43^Ca, ^47^Ti, ^51^V, ^52^Cr, ^53^Cr, ^55^Mn, ^56^Fe, ^57^Fe, ^59^Co, ^60^Ni, ^63^Cu, ^88^Sr, ^90^Zr, ^95^Mo, ^137^Ba, ^182^W and ^208^Pb. The ^13^C and ^29^Si signals were investigated as internal standards. All signals were collected using the ICP-MS standard mode (i.e., without using the collision cell) unless specified. Details on the working parameters are reported in Table 3.

Concerning the ablation pattern, two different approaches were applied depending on the sample preparation:

1. For the SiC tablets:
  - Line analysis (spot from 20 to 40 µm, length from 1 at 2 mm, scanning speed = 40 µm/s, frequency = 10 Hz and fluence from 1 to 15 J/cm^2^). The ablation was performed up to 5 times along the same lines in order to evaluate surface contamination or any depth profiles inside the material.
2. For the SiC powder embedded in epoxy resin:
  - Spot analysis was performed (spot size = 120 µm, frequency = 10 Hz, ablation time = 10 s and fluence = 3.8 J/cm^2^). The ablation was performed 3 times on the same spot and the third ablation was used for quantitative purposes. Five replicate analyses were performed on spot in randomized positions.
  - Line analysis was performed (spot size = 120 µm, length = 1 mm, scanning speed = 20 µm/s, frequency = 10 Hz and fluence = 3.8 J/cm^2^). The ablation was performed up to 5 times on the same line and the fifth line was used for quantitative purposes. Three replicate analyses were performed.

Regardless of the sample preparation and ablation pattern used, the mean values of the transient signals generated (after subtraction of the background signal) were calculated for all the elements: the obtained values were used for quantitative purposes. The instrumental sensitivity was estimated for each element by dividing the mean value obtained for BAM S003a by the concentration in mg/kg of the element considered. This calibration was carried out daily for each batch of analyses. The sensitivity was then used to calculate the concentrations in the SiC samples. All data elaborations were automatized by developing an Excel Macro to minimize the data elaboration time.

Finally, once the optimized sample preparation and ablation conditions were defined, the performance of the Nd: YAG laser operating at 266 nm was compared with that of a 193 nm ArF excimer laser ablation system (Analyte Excite, Teledyne, Thousand Oaks, CA, USA) equipped with a HelEx II Active 2-Volume Ablation Cell.

## 4. Conclusions

The goal of accurately quantifying trace elements in ultrapure SiC (5N, 99.999% purity target) was achieved in this work by developing a new analytical protocol for the direct analysis of the solid material with LA-ICP-MS.

Our results demonstrated the total unsuitability of the microwave-assisted acid digestion approach for such a pure target (sum of all contaminants <10 mg/kg) as this method is particularly critical for (i) extreme conditions (in terms of time and temperature) necessary for the complete dissolution of SiC, which potentially entail safety risks and damage to the vessels, also calling into question any advantage over the direct GD-MS technique, (ii) the contamination occurring during the digestion process and the inadequate purity of ultrapure acids (in particular H_2_SO_4_ and HF) and (iii) the very slow dissolution kinetics (in this work roughly 1 mg/h). This latter point is expected to be strongly dependent on the particle size distribution.

Differently, the developed LA-ICP-MS method (embedding in the epoxy resin of SiC without any pretreatment followed by LA-ICP-MS spot analysis) resulted as reliable and suitable for routine screening analysis as the order of magnitude of elemental concentrations was accurately determined, even when elements were present at the sub-ppm level. Importantly, such accuracy was verified independently of the grain size and crystallographic characteristics of the SiC materials.

Finally, it should be remarked that the extremely simplified sample work-up of the proposed LA-ICP-MS approach represents a significant advancement with respect to previous literature, also providing beneficial effects in terms of analysis costs, making the proposed protocol cheaper than GD-MS determinations.

Future studies will consider the optimization of the sample preparation (e.g., preliminary grinding by using a boron carbide mortar) and the introduction of a multi-point calibration technique (fully exploiting the size of the ablation chamber) to extend the number of elements to be quantified and to improve the accuracy of the proposed analytical protocol.

## Figures and Tables

**Table 1:** Concentrations determined for the 11 selected trace elements in samples SiC-1 and SiC-2 (in tablet form). LA-ICP-MS data were obtained by external calibration (BAM S003a as the single calibrant). Standard deviations of LA-ICP-MS data were calculated by determining the standard deviation of the transient signal. GD-MS data were obtained by using the Relative Sensitivity Factors (RSFs, see details in the experimental section).

Elements	SiC-1 (mg/kg)		SiC-2 (mg/kg)	
	GD-MS	LA-ICP-MS (Tablet)	GD-MS	LA-ICP-MS (Tablet)
Na	0.6 ± 0.1	1.9 ± 0.7	0.33 ± 0.07	0.4 ± 0.2
Mg	0.07 ± 0.01	0.9 ± 0.1	0.28 ± 0.06	0.47 ± 0.05
Al	11 ± 2	16 ± 7	10 ± 2	8 ± 4
Ca	<0.2	25 ± 2	3.5 ± 0.7	6.2 ± 0.9
Ti	1.3 ± 0.3	3.8 ± 0.8	2.5 ± 0.5	4.5 ± 0.9
Cr	<0.3	0.5 ± 0.1	<0.3	0.5 ± 0.2
Mn	<0.05	0.12 ± 0.01	<0.05	0.1 ± 0.03
Fe	0.42 ± 0.08	13 ± 3	4.6 ± 0.9	9 ± 3
Ni	<0.05	1.4 ± 0.6	25.3 ± 0.5	11 ± 4
Cu	<0.05	0.5 ± 0.2	<0.05	0.2 ± 0.4
Zr	0.19 ± 0.04	1.9 ± 0.4	0.7 ± 0.2	1 ± 2

**Table 2:** Limit of detection estimated for the 13 selected trace elements. The LOD was estimated by using the calibration sensitivity determined using the standard reference material BAM S003a.

Elements	LOD (mg/kg)
B	0.18
Na	0.13
Mg	0.001
Al	0.036
Ca	0.55
Ti	0.084
V	0.039
Cr	0.15
Mn	0.10
Fe	0.85
Ni	0.11
Cu	0.09
Zr	0.004

**Table 3:** ICP-MS operating parameters used for all the ICP-MS and LA-ICP-MS measurements.

ICP-MS Parameters	
RF power (kW)	1.55
Auxiliary gas flux (L/min)	0.8
Cooling gas flux (L/min)	14
Makeup gas flux (L/min)	0.9
Dwell time (ms)	10

## Data Availability

Not applicable.

## Supplementary Materials

The following supporting information can be downloaded at: https://www.mdpi.com/article/10.3390/molecules28062845/s1, Details on microwave digestion experiments; Figure S1: microwave-assisted digestion system configuration; Figure S2: SEM images of SiC samples after and before microwave treatment; Figure S3: XRD pattern of SiC-1 after thermal treatment; Figure S4: picture of a BAM S003a sintered tablet after laser ablation; Figure S5: picture of SiC-3 and SiC-4 samples embedded in the epoxy resin; Figure S6: XRD pattern of SiC samples; Figure S7: temperature program used for microwave-assisted acid digestion of SiC samples. Table S1: Trace element concentrations in ultrapure acids before and after acid digestion; Table S2: GD-MS analysis of BAM S003a; Table S3: GD-MS analysis of SiC samples; Table S4: LA-ICP-MS analysis of SiC samples.

## Sample Availability

Samples of the compounds are available from the authors.
