# Supplementary material for: Tackling the Challenging Determination of Trace Elements in Ultrapure Silicon Carbide by LA-ICP-MS
Source: Molecules. 2023 Mar 21;28(6):2845. doi: 10.3390/molecules28062845 (PMC10051955; doi:10.3390/molecules28062845)
Supplement: Supplementary file 1 [file molecules-28-02845-s001.zip › molecules-2212907-supplementary.pdf]

Supplementary Material

Microwave-assisted acid digestion experiments

The suitability of acid digestions was preliminary investigated using harsh conditions reported in the literature as a starting point (i.e., using a mixture H_2SO_4 + HNO_3 + HF at 230°C for several hours [16]). A "Vessel-inside-Vessel" microwave technique was initially utilized (for details see the experimental section), since this strategy limits as much as possible the dilution factor and the occurrence of contamination, especially during HF removal [41].

In the above reported conditions, even after a 48h digestion, the mineralization of 50 mg of SiC was not complete. Importantly, it was also observed the deformation of the outer PTFE vessels and the partial fusion of the inner PFA ones. Both these evidences highlight the unsuitability of the "Vessel-inside-Vessel" configuration to perform acid digestion under extreme temperature conditions. Such a failure is probably due to the inaccurate temperature control, which is always performed in the scavenging solution, rather than in the sample (Figure 1a): the different absorption efficiency of the microwaves from the inner acid mixture and the outer scavenging water may account for the uncontrolled process and the consequent melting of the polymeric vessels (see [42] for more details on such a configuration).

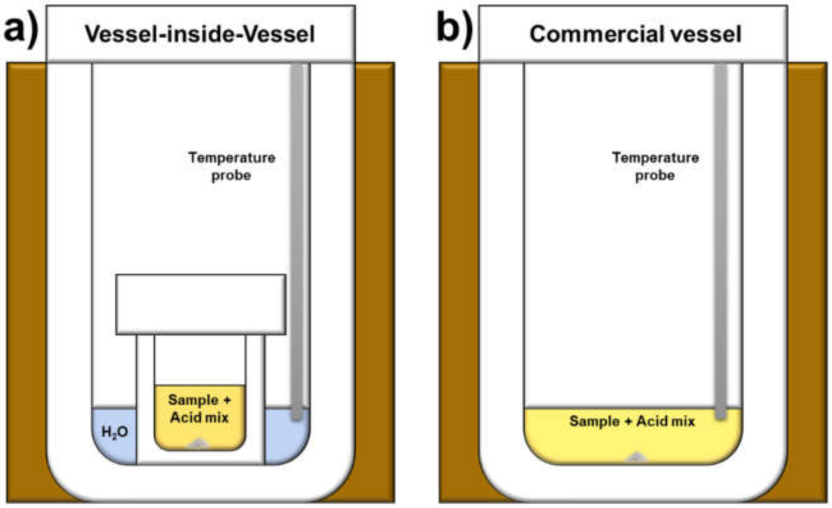

Figure S1. Schematic sketch of the two configurations used for microwave-assisted acid digestion: (a) "Vessel-inside Vessel" and (b) conventional PTFE vessel. See the experimental section for additional details.

We therefore decided to use a conventional configuration to ensure a more accurate temperature control (see Figure 1b). After some preliminary tests, three different amounts of sample (45.4 mg, 54.1 mg and 66.2 mg of SiC) were treated aiming at investigating the dependence of the digestion process on the mass of the solid sample. At the end of a 48 h digestion the complete dissolution of the sample was observed only for the 45.4 mg sample. These observations highlight the extremely slow mineralization kinetics, with an estimated digestion rate around 1 mg/h, and the need of an accurate temperature control to properly work in harsh conditions to avoid to locally exceed the vessels' deformation temperature. Scanning Electron Microscopy (SEM) analysis confirmed the slow mineralization rate: as can be clearly seen in Figure 2, the dimensions of residual SiC particles gradually decrease during the digestion process.

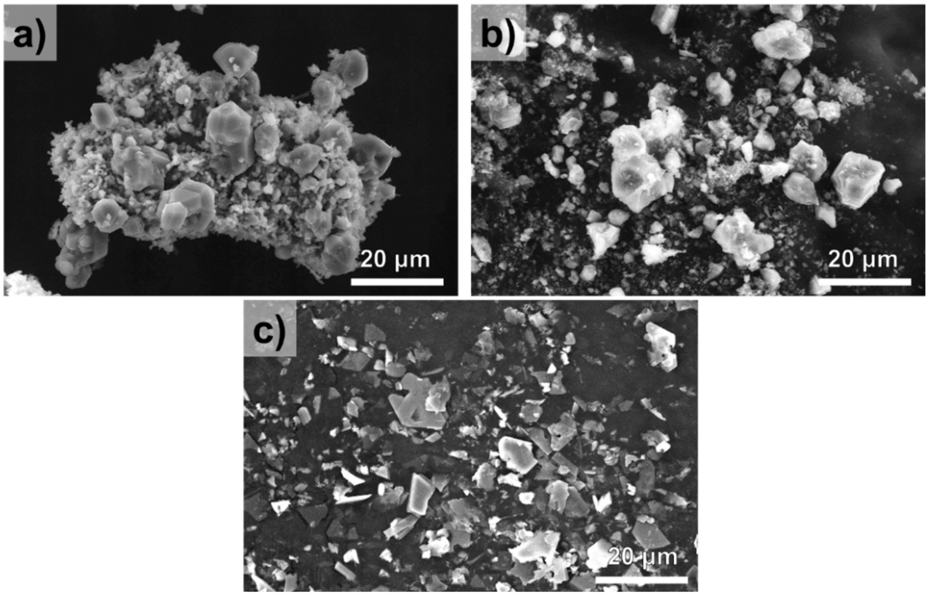

Figure S2. SEM images of a SiC sample (51.4 mg) (a) not treated, (b) after 24 hours and (c) 48 hours.

At this point, before trying to test harsher and more efficient digestion conditions, we decided to investigate if the purity of blank acid solutions and/or the extent of their contamination during microwave digestion is sufficient to perform the determination of elemental impurities down to the sub-mg/kg in SiC. It is in fact well know that the porosity of PTFE vessels produces strong memory effects that cannot be easily erased, especially for non-volatile species [43].

Table S1. Concentrations (expressed in µg/kg) of the trace elements determined for the acids (and their mixture) before and after acid digestion. All analyzed solutions were prepared and diluted to 30 g and again 1:10 (see experimental section).

**Elements**	**Concentration (µg/kg) in diluted solutions**					
	**Instrumental LOD**	**HNO_3**	**HF**	**H_2SO_4**	**HNO_3+HF +H_2SO_4**	**HNO_3+HF +H_2SO_4 (after MW digestion)***
B	0.3	0.7 ± 0.2	1.8 ± 0.3	0.3 ± 0.2	1.0 ± 0.2	60 ± 10
Na	1.3	6.1 ± 0.8	9.5 ± 0.7	12 ± 1	14.3 ± 0.9	27 ± 4
Mg	0.02	2.8 ± 0.09	1.42 ± 0.05	5.9 ± 0.7	9.5 ± 0.4	22 ± 2
Al	0.1	1.52 ± 0.09	0.94 ± 0.06	39 ± 1	18 ± 1	25 ± 2
Ca	0.09	2.8 ± 0.1	3.4 ± 0.2	1.1 ± 0.1	5.9 ± 0.3	33 ± 2
Ti	0.007	0.23 ±0.02	0.92 ± 0.09	0.53 ± 0.02	2.3 ± 0.2	2.7 ± 0.5
V	0.005	0.008 ± 0.006	2.0 ± 0.2	0.024 ± 0.001	2.7 ± 0.1	0.9 ± 0.1
Cr	0.02	0.93 ± 0.04	1.6 ± 0.2	0.21 ± 0.06	1.3 ± 0.1	2.6 ± 0.4
Mn	0.003	0.039 ±0.005	0.06 ± 0.01	0.04 ± 0.02	0.09 ± 0.01	0.27 ±0.09
Fe	0.02	5.2 ± 0.2	6.3 ± 0.4	4.9 ± 0.4	7.9 ± 0.1	23 ± 4
Co	0.002	0.003 ± 0.001	0.008 ± 0.005	0.004 ±0.002	0.014 ±0.003	0.031 ± 0.008
Ni	0.006	0.284 ± 0.003	10.5 ± 0.2	0.86 ± 0.02	18 ± 1	14 ± 1
Cu	0.005	0.22 ± 0.03	0.10 ± 0.01	0.44 ± 0.02	0.8 ± 0.1	1.6 ± 0.2
Zn	0.02	1.1 ± 0.2	2.5 ± 0.3	3.1 ± 0.1	7.2 ± 0.2	14 ± 5
Zr	0.0004	0.012 ± 0.002	0.091 ± 0.002	0.04 ± 0.01	0.12 ± 0.01	0.34 ± 0.01
Ag	0.001	0.022 ± 0.002	0.05 ± 0.02	0.012 ± 0.007	0.21 ± 0.06	0.06 ± 0.04
Cd	0.0003	0.0004 ± 0.0003	0.03 ± 0.01	0.0011 ± 0.0009	0.0342 ± 0.0003	0.022 ± 0.006
Sn	0.02	0.02 ± 0.01	0.05 ± 0.03	0.24 ± 0.07	0.2 ± 0.1	0.5 ± 0.1
Ba	0.004	0.009 ± 0.005	0.25 ± 0.02	0.02 ± 0.01	0.27 ± 0.03	0.41 ± 0.08

Pb	0.004	0.233 ± 0.006	0.63 ± 0.02	0.082 ± 0.005	0.9 ± 0.1	1.5 ± 0.2
W	0.001	0.001 ± 0.001	0.001 ± 0.001	0.001 ± 0.001	0.001 ± 0.001	0.08 ± 0.05

*A vessel cleaning cycle is performed in the same conditions before evaluating the contamination induced by the digestion procedure.

From the data reported in Table 1 we can immediately see that the concentration of most of the elements in blank acid solutions increases after the mineralization program, while some of them are already quite high in pristine pure acids. Taking into account that a dilution factor of at least 6000 (the dilution factor is referred to the SiC mass) must be applied in the wet digestion procedure (to reduce the sulfate loading), a concentration of 1 μg/kg in the analyzed solution correlates to 6 mg/kg in the SiC powder. Indeed, these evidences clearly indicate that such approach is not suitable for the analysis of highly pure 5N SiC samples, where the sum of all contaminants must not exceed 10 mg/kg.
Therefore, from the data above reported, we concluded that the acid digestion technique is not suitable for the determination of impurities in highly pure 5N SiC samples, and that it is very difficult (or impossible) to foresee significant improvements of this technique to overcome the above reported problems. Rather, a direct analytical method, with a very limited sample workup, should be exploited. For these reasons it was decided to deeply exploit the potentialities of the LA-ICP-MS technique as a cheaper alternative to GD-MS (considered the reference technique in this field), for the direct analysis of solid SiC without the need of wet-based pretreatments.

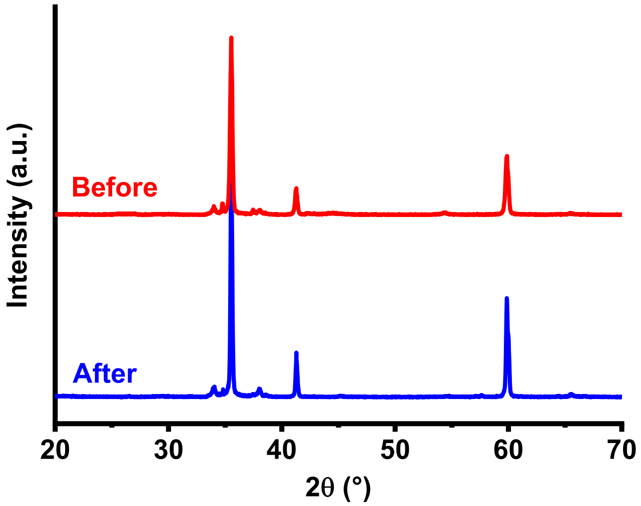

Figure S3. XRD pattern of sample SiC-1 before and after the thermal treatment at 1000°C for 2 hours.

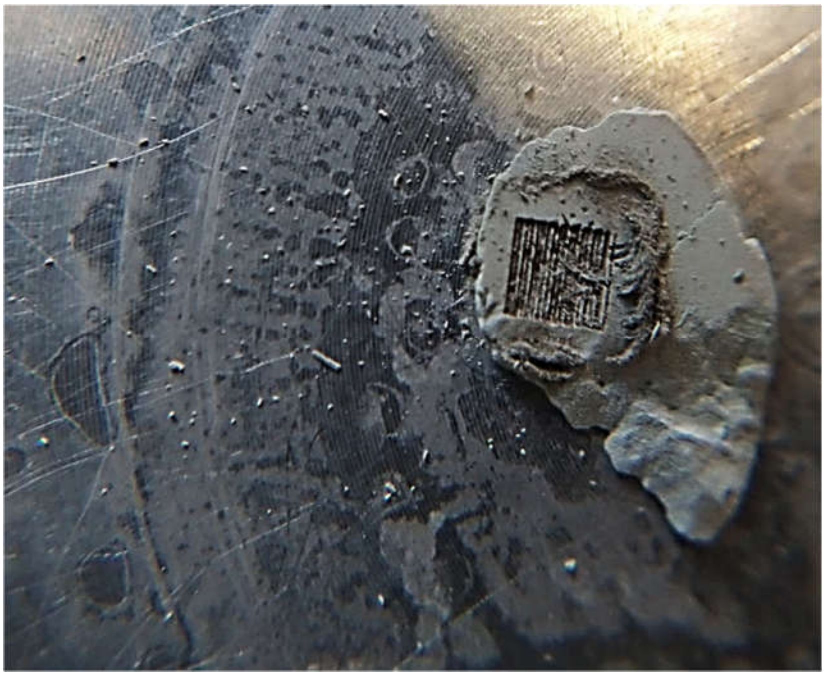

Figure S4. Picture of sample BAM S003a (a part of the sintered tablet) inside the ablation chamber after several line analysis.

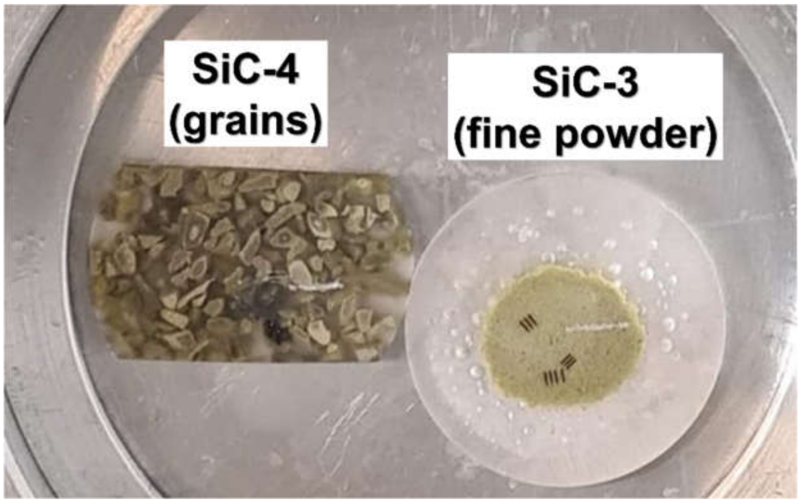

Figure S5. Picture of SiC-3 and SiC-4 samples directly embedded in the epoxy resin. Well-defined ablation lines are visible for sample SiC-4.

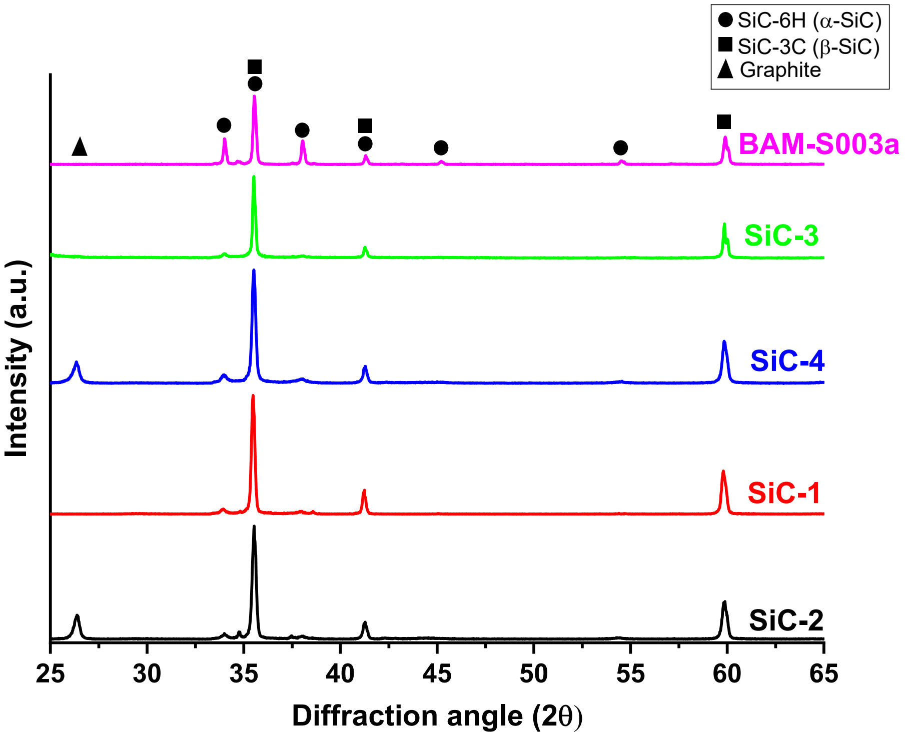

Figure S6. XRD pattern of SiC samples analyzed in this work.

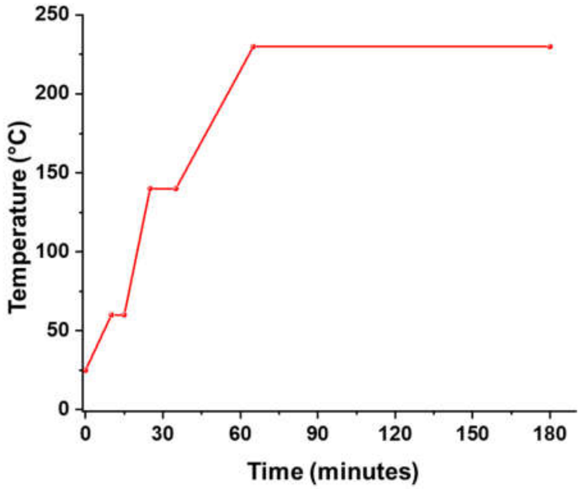

Figure S7. First three hours of the temperature programm used for microwave-assisted acid digestion of SiC samples.

Table S2. Concentrations determined for 13 selected trace elements in BAMS003a by GD-MS. The certified values (according to the Bundesanstalt für Materialforschung und -prüfung (BAM) certificate) are the means of up to 22 series of results obtained by different laboratories using up to 8 different analytical methods.

Elements	BAMS003a (mg/kg)	
	GD-MS	Certificate
B	60 ± 11	63 ± 7
Na	15 ± 3	17.7 ± 0.8
Mg	6 ± 1	6.3 ± 0.9

Al	400 ± 80	372 ± 20
Ca	25 ± 5	29.4 ± 2.7
Ti	70 ± 10	79 ± 4
V	50 ± 10	41 ± 5
Cr	3.2 ± 0.6	3.5 ± 0.4
Mn	1.5 ± 0.3	1.44 ± 0.25
Fe	160 ± 30	149 ± 15
Ni	37 ± 7	32.9 ± 2.7
Cu	1.3 ± 0.3	1.5 ± 0.4
Zr	24 ± 5	25.2 ± 2.0

Table S3. Concentrations determined for selected trace elements in SiC samples by GD-MS. Elements not included in this table were not quantified as they are below the limit of detection of the GD-MS technique (0.05 mg/kg for most elements) for all the samples.

Elements	GD-MS (mg/kg)			
	**SiC-1**	**SiC-2**	**SiC-3**	**SiC-4**
Na	0.6 ± 0.1	0.33 ± 0.07	0.9 ± 0.2	0.043 ± 0.008
Mg	0.07 ± 0.01	0.28 ± 0.06	0.21 ± 0.05	< 0.05
Al	11 ± 2	10 ± 2	16 ± 3	7 ± 1
Ca	< 0.2	3.5 ± 0.7	4.5 ± 0.9	< 0.2
Ti	1.3 ± 0.3	2.5 ± 0.5	3.2 ± 0.6	0.5 ± 0.1
Fe	0.42 ± 0.08	4.6 ± 0.9	2.2 ± 0.4	0.17 ± 0.03
Ni	< 0.05	25 ± 5	17 ± 3	0.11 ± 0.02
Zr	0.19 ± 0.04	0.7 ± 0.2	0.6 ± 0.1	0.14 ± 0.03

Table S4. Concentrations determined for selected trace elements in SiC samples by LA-ICP-MS. Uncertainties are express as one time the standard deviation estimated over five replicate analyses.

Elements	LA-ICP-MS (mg/kg)				Precision
	**SiC-1**	**SiC-2**	**SiC-3**	**SiC-4**	**RSD%**
Na	< 0.13	1.17 ± 0.03	0.65 ± 0.02	< 0.13	2.7
Mg	0.140 ± 0.003	0.92 ± 0.02	0.64 ± 0.04	< 0.001	3.3
Al	9.2 ± 0.4	25 ± 2	25 ± 3	12.4 ± 0.3	6.8
Ca	< 0.55	13.3 ± 0.7	15.1 ± 0.9	< 0.55	5.8
Ti	0.92 ± 0.06	2.3 ± 0.3	2.9 ± 0.4	1.38 ± 0.09	9.7
Fe	1.07 ± 0.01	3.53 ± 0.04	5.1 ± 0.1	0.86 ± 0.01	1.5
Ni	< 0.11	8.3 ± 0.1	32.9 ± 0.3	< 0.11	1.1
Zr	0.75 ± 0.01	1.02 ± 0.01	1.74 ± 0.03	1.10 ± 0.01	1.2
